# Social and financial efficiency: Institutional characteristics of the partner organizations of Pakistan Poverty Alleviation Fund

**DOI:** 10.1371/journal.pone.0244444

**Published:** 2020-12-28

**Authors:** Zulfiqar Ali, Ikram Ullah, Naila Nazir, Muhammad Asif, Muhammad Azeem

**Affiliations:** 1 Govt College Peshawar, Peshawar, Pakistan; 2 Department of Economics, University of Peshawar, Peshawar, Pakistan; 3 Department of Economics, University of Malakand, Chakdara, Pakistan; 4 Department of Statistics, University of Malakand, Chakdara, Pakistan; Shandong University of Science and Technology, CHINA

## Abstract

Efficiency analysis of the Partner Organizations can benefit all the microfinance sector's key stakeholders to benchmark the current scene and formulate optimal policy agenda. This study seeks to measure the partner organizations of the Pakistan Poverty Alleviation Fund's social and financial efficiency and to identify causes and sources of their inefficiencies. A non-parametric technique known as Data Envelopment Analysis is applied to investigate the Partner Organizations' efficiency throughout 2005–2015. The required data was obtained from the database of the Mix-Market and Pakistan Microfinance Network. The social and financial efficiency was estimated assuming Constant Return to Scale, Variable Return to Scale, and with respect to the Operational Scale of the Partner Organizations. Results revealed that the partner organizations are more scale efficient (median = 75%) than pure technically efficient (median = 55%). Further, graphical representations show a decreasing linear trend and negative serial correlation in the percentage of efficient partner organizations. The model fit results show that institutional characteristics that influence partner organizations' efficiencies significantly include their age, Operational Self-Sufficiency, personnel, loan officers, assets and debt. Finally, the diagnostic tests for endogeneity, heteroskedasticity, heterogeneity, and cross-sectional dependence were performed.

## 1 Introduction

Poverty alleviation is one of the major global challenges. It is evident, particularly in developing countries, including Pakistan, where various measures have been initiated to curb poverty. Pakistan is a developing country and is home to approximately 55 million (approximately 25 percent of the total population) people living below the national poverty line, and 46 million (20.5 percent of the total population) are faced with undernourishment [1]. Further, the country ranks in the lowest quartile of the Human Development Index [2]. As a result, Pakistan attempted to start various poverty alleviation programs over the years. For instance, the Benazir Income Support Program (BISP), Waseela-e-Taleem, Pakistan Baitul Mal (PBM), Workers Welfare Fund (WWF), Employees Old-Age Benefits Institutions (EOBI), and Pakistan Poverty Alleviation Fund (PPAF). The country showed progress in Human Development; however, the progress is unsatisfactory compared to the other South Asian countries [3].

Poverty alleviation is on the core agendas of the government since the very beginning. During the 1960s, the establishment of the Agricultural Development Bank of Pakistan (which was later renamed to Zarai Taraqiati Bank Limited) to provide soft loans to the society's rural and marginalized segments was the first attempt of this endeavor. Pakistan established two formal microfinance institutions, Orangi Pilot Project and Agha Khan Rural Support Program (currently known as First Micro Finance Bank), following the Grameen Bank's success in the early 1980s. The encouraging experience of these two institutions triggered the establishment of other Non-Governmental Organizations (NGOs) for the purpose, resulting in the establishment of the Pakistan Microfinance Network (PMN) in 1998 [4].

Grameen Bank was established in 1976 has drawn global attention by providing microcredit to the marginalized people of rural Bangladesh. Professor Dr. Muhammad Younis and Grameen Bank were awarded the Nobel Peace Prize, in 2006, for their struggle towards the poor masses' socio-economic uplift. Their microfinance has been globally recognized as a vital liberating force and an important instrument in the fight against poverty. The revolution of microfinance than got momentum and due to the frontline role of Grameen Bank. The Bank performed an instrumental role against poverty by providing microcredit to the poor masses to create self-employment opportunities [5].

The Grameen Bank model has globally been replicated and got fame in the microfinance sector. According to the model, a careful selection of the targeted group of extreme poor is recommended after the field officer's approval. The group shares the same socio-economic class. The Bank does not require any financial or physical collateral. After necessary legal formalities, the bank advance loan to the group of people usually contains five members. The Bank uses social pressure as an alternative of collateral, where every member in the group is responsible for the repayment of the other members' loan. Most of the African, South, and Southeast Asian countries follow the Grameen Bank Model. The fundamental reasons behind the success of Grameen Bank are its distinct and decentralized organizational structure, client-oriented delivery system, organizational culture, and its internal environment [6].

Recently, Pakistan has established the Pakistan Poverty Alleviation Fund (PPAF henceforth) with the World Bank and Agricultural Development Bank of Germany. The PPAF provides subsidized funds to more than 50 percent of the Microfinance Partner Organizations (henceforth referred MFIs) and has a total market share of 44 percent in the disbursement of microcredit Pakistan [7, 8]. Despite the establishment of various microfinance institutions practicing for almost 40 years, statistics mentioned in the opening paragraph show the country's utter failure to achieve the desired poverty alleviation levels. It is also evident from a State Bank of Pakistan [9] and some research studies such as [10–16]. These studies show inefficiencies in Microfinance Institutions (MFIs) to be one of the main reasons for their dismal performance. As a result of the coronavirus pandemic's current outbreak, poverty is expected to double [17, 18]. In this regard, the role of the MFIs is important in the fight against poverty and malnutrition.

It is worth knowing that MFIs have a dual role, social as well as financial. To operate independently and effectively on a large scale, examining the social and financial efficiency of the MFIs is highly important. Studies, such as [19], reported that the assessment of social and financial of the MFIs is imperative for optimal policy agenda. Among the others, one of the key reasons for social and financial efficiency analysis is the withdrawal of the funds by the donors from the microfinance sector, which compel the management of MFIs to priorities social and financial efficiency of the institutions [20]. Therefore, a fresh and thorough assessment of the financial and social efficiency levels of the POs/MFIs, and the institutional determinants of the two types of efficiencies is highly desirable and timely. Hence the present study is designed to measure the level of financial and social efficiency of the POs/MFIs and identify important determinants of the two types of efficiencies. Further, it is investigated, graphically, how the percentage of efficient POs/MFIs varies with time. The rest of the paper is organized as follows—section 2 reviews relevant recent literature from Pakistan and the rest of the world. Section 3 outlines the data and methodology used in the paper. The study results are given in section 4 with subsequent discussion, and section 5 concluded the paper.

## 2 Relevant literature

Table 1 summarizes a representative list of recent research studies on the microfinance industry's efficiency conducted in the rest of the world. A similar summary of the empirical studies targeting either efficiency or sustainability of Pakistan's MFIs is given in Table 2. Besides the studies listed in Table 1, other recent studies that use multinational samples to investigate the social and financial efficiency of the MFIs include [21–24]. However, none of these studies found their sample of MFIs to be producing on the efficient frontier.

**Table 1:** Recent empirical literature from the rest of the world.

Study	Sample & MFIs Origin	Efficiency Measure(s)	Empirical Findings	
			Efficiency Levels	Correlates of Efficiency/Sustainability
[25] *	2010–2014	Gross Loan Portfolio	Only about 10% of the sample MFIs are technically efficient, and 20% are pure technically efficient.	(-) Women in BoD, (-) Profitability, (-) Credit Risk, (-) Size, (-) OSS, (+) Age, (+) Outreach, (+) Financial Intermediation, (+) Level of Country’s Development.
	(418 MFIs)	Active Borrowers		
	Multinational			
[26]*	2005–2014	Gross Loan Portfolio	The efficiency scores ranged from 54% to 74 during the study period.	(+) New & Young MFI, (-) Ownership Type (NGO), (+) Debt to Equity Ratio, (+) Branches, (+) Crisis.
	(99 NMFIs)			
	Multinational	Active Borrowers		
[27]	2007–2013	Gross Loan Portfolio	For-Profit MFIs are more financially efficient (FP = 75% > NP = 50%) while Non-for-Profit MFIs are more socially efficient (NFP = 68% > FP = 47%).	**Non-Profit MFIs (NP):** Financial inefficiency = (-) Size & Social Inefficiency = (+) Size.
	(162 MFIs)	Women		
	Multinational	Borrowers		**Profit MFIs (FP):** Financial inefficiency = (-) age & Social Inefficiency = (-) Subsidies, (+) Size
[28]	2011	Gross Loan Portfolio	The average financial efficiency & social efficiency in the sample MFIs is found to be 94.15% and 73.75%, respectively.	**Financial Efficiency**: (+) Age, (-) Operational Expenses, (+) Cost per Borrower.
	(28 MFIs)	Financial Revenue Outreach		**Social efficiency**: (+) Staff Productivity, (-) Cost per Borrower
	Vietnam			
[29]*	2007–2012	Financial Revenue	Due to improvements in technical efficiency, on average, 2.1% improvements per annum takes place in the total factor productivity of the South Asian MFIs.	(+) Returns on Assets, (-) Total Asset, (-) Debt to Equity Ratio, (-) Cost per Loan
	(50 MFIs)	Active Borrowers		
	South Asia	Average loan Balance/ GNI		
		Per Capita		
[30]	2009–2014	Loan Outstanding	The average efficiency scores in the sample MFIs range between 0.63 and 0.67.	(+) Average Loan Balance, (+) Number of Clients, (+) Returns on Asset, (-) Age, (-) Interest Caps (dummy).
	(122 MFIs)	Deposits		
	Bangladesh			
[31]*	2014–2016	Gross Loan Portfolio	The average efficiency scores range between 56.50% and 68.90% in the sample MFIs	(+) Average Loan Balance, (+) Female Borrowers, (+) Borrowers per Staff, (+) OSS, (+) Yield on Gross Portfolio, (-) Cost per Borrower
	(28MFIs)	Total revenue Women Borrowers		
	India & Bangladesh			
[21]*	2013	Gross Loan Portfolio	Regardless of reference frontier, the average efficiency scores, both social and financial, of the MFIs are found to be extremely low.	**Social Efficiency:** (-) Age, (+) size, (-) return on assets, (-) debt to equity ratio
	(420 MFIs)	Financial Revenue,		
	Multinational	Active Borrowers		
				**Financial Efficiency:** (+) Age, (+) size, (+) return on assets

**Note:** For the studies marked as (*), column 3 includes only a subset of these studies' output variables.

**Table 2:** Recent literature summary from Pakistan.

Study	Sample	Efficiency Measure(s)	Empirical Findings	
			Efficiency Levels	Correlates of Efficiency/Sustainability
[36]	2007–2013 (148 MFIs)	Financial Margin Gross Loan Portfolio	The scores of TE ranged from 60 to 90, implying that none of the MFI produced on the efficient frontier.	(+) Age of MFIs, (+) No. of Branches, (+) No. of Staff, (-) Average Loan Balance
[10]	2008–2009 (170 MFIs)	No. of Female Borrowers	On average, 20% of the sample MFIs are inefficient.	(+) Borrowers per Staff, (+) Age of MFIs, (+) Small Loan Portfolio
		Returns on Assets		
[11]	2006–2016 (15 MFIs)	Gross Loan Portfolio	Efficiency improving overtime but 14 out of 15 of the MFIs investigated performed below the efficient frontier in both the periods (2007 & 2016).	NA
		No. of Active Borrowers		
[37]	2011–2015 (32 MFIs)	Financial Self-Sufficiency	NA	(+) Size of MFIs, (+) Loan Portfolio/Total Assets, (-) Portfolio at Risk, (-) Breadth of Outreach, (-) Management inefficiency, (-) Operating cost ratios.
[12]	2014 (38 MFIs)	Financial Income Operational Income	26 out of the 38 sample MFIs operated below the efficient frontier.	NA
[38]	2005–15 (10 NGOs)	Financial Self-Sufficiency	NA	(+) Depth of Outreach, (+) Staff Productivity, (-) Cost per Borrower.
[39]	2004–2015 (10 NGOs)	Operational Self-Sufficiency	NA	No trade-off between outreach and sustainability.
[13]	2000–2017 (28 MFIs)	Depositors/Staff Member	16 out of 28 sample MFIs are found to be operationally inefficient.	NA
		Loan Balance/Female Borrower		
[14]	2008–2014 (05 MFIs)	Active Borrowers	Pakistani MFIs are less financially and operationally efficient as compared to the South Asian average.	NA
		% of Female Borrowers		
[16]	2019 (22 MFIs)	Revenue/Assets	20 out of 22 sample MFIs are inefficient.	(+) Age of MFIs, (+) Size of MFIs, (+) Number of Inputs
		Female Borrowers		
[15]	2010–2016 (22 MFIs)	Financial Revenue	8 out of 22 sample MFIs are socially and financially inefficient.	NA
		Female Clients		
[40]	2008–2014 (29 MFIs)	Operational Self-Sufficiency	NA	(+) MFIs' size, (-) Cost Inefficiency, (-) Portfolio at Risk, (-) Average Loan Size, (+) Yield on Loan Portfolio

It is worth noticing from Table 1 that most of the recent literature has focused on multinational samples. This practice is good to a certain extent for enabling a more precise estimation of the parameters of interest and identifying differences across regions. However, on the other hand, such practice has overlooked heterogeneities presented at the MFIs level. For instance, using a multinational sample of 162 MFIs, the study [27] reported financial and social efficiencies, approximately 75% and 68%, respectively. In contrast, using a single country's sample, Kaur [32] reported the financial efficiency of the Indian MFIs to be about 84% and social efficiency of about 32% [33]. Similar findings are also reported by some other studies using a single country sample, for example, Van Damme et al. [34] and Efendic and Hadziahmetovic [35] for Sri Lanka and Bosnia-Herzegovina, respectively.

Recent relevant literature from Pakistan can be divided into four groups. The first group contains empirical literature that investigates various determinants of the financial sustainability of the MFIs in Pakistan. For example, studies [37, 38, 40]. These studies' results are in consensus regarding the positive impact of the size of the MFI, loan portfolio, staff productivity/management efficiency, and the yield on loan portfolios over the financial sustainability of the MFIs in Pakistan. Portfolio at risk and operating costs contributed negatively, while results regarding the outreach measures showed a mixed relationship with the financial sustainability of the MFIs.

The second group of studies includes literature investigating the trade-off between financial and social (outreach) efficiency. Examples of such studies are [14, 39, 41]. It is worth noticing that not all studies show evidence of a trade-off between social and financial efficiency. For instance, the studies [14, 39] found no evidence of a trade-off between social and financial efficiency. In contrast, the study of Ullah et al. [41] found a clear trade-off between the two efficiency measures. The study [41], however, included the MFIs from all six regions of the world in the Sample.

In the third group, those studies, for example [11–13, 36], are included in which the financial efficiency of the MFIs in Pakistan was measured but not social efficiency. These studies reported that most Pakistani MFIs are producing below the financially efficient frontier, using various financial performance measures. For instance, Financial Margin & Gross Loan Portfolios were used by Riaz & Gopal [36], Gross Loan Portfolio and Number of Active borrowers by Ahmed et al. [11], Financial and Operational Income by Farooq [12], and Depositors per Staff and Average Loan Balance per Female Borrower were used by Khan et al. [13] to measure financial efficiency. Further, financial efficiency scores are reported to be improving over time [11]. Finally, the fourth group of recent studies includes those in which the social and financial efficiency of the MFIs were measured. The most notable amongst this group are [10, 15, 16]. Akram et al. [10] measured financial and social efficiency by ‘returns on assets’ and the ‘proportion of female borrowers’. They reported approximately 20% of the MFIs in the South Asian region are technically inefficient. The study also reported that the main determinants of the efficiency of MFIs in the South Asian region were 'write off ratio', 'small loan portfolio', 'age of the MFI', and 'borrowers per staff', using the Tobit regression specification. It is worth knowing that these results may not be generalized to the normal period as the data include the period of financial crises 2008–09.

Likewise, Iqbal et al. [16] measured the social and financial efficiency of a sample of 22 MFIs in Pakistan. They measured financial efficiency with financial revenue to assets ratio and the social efficiency by the number of female borrowers. The input variables of the study are total assets, the number of employees, and operating expenses. The study showed that none of the sample MFIs were efficient by utilizing single input, using the DEA method. Further, increasing the number of inputs improves the efficiency of the MFIs. Moreover, the age and size of the institution also contributed substantially to the efficiency of MFIs. Similarly, Mohsin et al. [15] also found 8 out of the 22 sample MFIs to be socially and financially inefficient.

The findings from the literature reviewed above are, however, not suitable for policy simulations in the local context. Given the cultural and institutional differences across countries—which are ignored in many studies based on multinational samples—international insights seldom help provide local policy content. The recent endogenous empirical literature, reviewed above, has several undesirable characteristics that make their results non-reliable. For instance, to highlight a few of the shortcomings, Table 3 enlist six recent representative studies. Three of the listed studies concern only the financial efficiency of the MFIs in Pakistan. Besides methodological issues discussed later in this study, Ahmad et al. [11] consider 'loan amount distributed' as an input and 'gross loan portfolio' as an output in their DEA specification. Technically, the two variables differ only by the number of 'write-offs' and, therefore, cannot be both input and output simultaneously. Likewise, the choice of input/output variables in these studies is also random. For instance, the studies [11, 12] ignores the importance of 'total assets' of the MFIs as an input. In literature, little work may be found that does not use assets as an input. Further, the use of 'average loan size' as an output variable by Mohsin et al. [14] is debatable.

**Table 3:** Discrepancies in the use of input/output variables.

Author(s)	Variables	
	As Inputs	As Output
Ahmad et al. [11]	***Loan Amount Disbursed***	***Gross Loan Portfolio***
	Total Staff	No. of Active Borrowers
	No. of Offices	
Farooq [12]	Financial Expenses	Financial Income
	Operating Expenses	Operating Income
Iqbal et al. [16]	Assets	Financial revenue/Assets
	Operating expenses	Average loan balance per borrower
	Employees	No. of female borrowers
Mohsin et al. [15]	Assets	Financial Revenue
	Operating Expenses	% of female clients
	Personal's	Average Loan Size
Akram et al. [10]	Assets	No. of Female Borrowers
	Personnel's	Gross Loan Portfolio
	Operating Expenses	Outstanding Loans
		Return on Assets
Riaz and Gopal [36]	Cost per Borrower	Financial Margin
	Financial Expenses	***Gross Loan Portfolio***
	Total Assets	

## 3 Data and methodology

### 3.1 Data and variables

This study's data is retrieved from the Mix-Market (http://www.mixmarket.org) and the PMN databases for the period 2005–2015. The number of POs / MFIs each year, however, vary. In the sample data, the minimum and maximum number of POs are 14 and 35 for 2005 and 2014. In this paper, the input and output variables have been selected based on existing literature. There are two broad approaches to the selection of input and output variables. These are the intermediation approach and the production approach. The intermediation approach considers funds acquired through loans and deposits as inputs while advancing as outputs [42, 43]. In contrast, the production approach considers an MFI like a production unit that utilizing many inputs at the same time to produce output [44, 45]. Since all the POs/MFIs in the sample receive subsidized funds from the PPAF, the intermediation approach–having the focus on mobilizing savings and accepting deposits—for selecting inputs and outputs is not appropriate. Hence the selection of inputs and outputs for this study is based on the production approach, which suits the context of the POs/MFIs selected in the sample [46, 47]. Hence the study utilizes operating costs, loan officers, and total assets as inputs while financial revenue, outreach, and the number of female borrowers as outputs. The selected input variables represent institutional features, costs, and assets structure of the POs. The output variables represent the financial (revenue side) and social mission (outreach and female borrowers) of the POs.

In Table 4, the input and output variables are listed and described. Likewise, to investigate covariates of the financial and social efficiencies of the POs, this study considers age, the number of branches, personnel, loan officers, operational self-sufficiency, total assets, and total debt outstanding as independent variables. All these variables represent the institutional characteristics of the POs that are expected to determine their social and financial efficiency. Table 5 below describes these institutional characteristics of the PO/MFIs and highlight their hypothesized relationship with efficiency.

**Table 4:** Description of input and output variables selected for DEA.

Type	Variable	Variable definition	Unit	Source
Input	Total Assets	Total Net Assets	PKR	MIX-market
Input	Operating Cost	The expenditures on salaries, rent, utilities, transportation, office supplies, and depreciation.	PKR	PMN
Input	Loan Officers	Number of active disbursement and collection staff	Number	MIX-market
Output	Financial Revenue	Revenue generated from the gross loan portfolio, investments & other operating revenue	PKR	PMN
Output	Outreach	The number of active borrowers.	Number	PMN
Output	Female Borrowers	Number of active female borrowers	Number	MIX-market

**Table 5:** Determinants of efficiency.

Variable	Measure	Unit	Hypothesized Relationship
**Super Efficiency**	Output of DEA	DEA Output	Dependent
**Number of Branches**	Number of Offices (including head office)	Number	(+)
**Personnel**	Number of employees	Number	(+)
**Loan Officers**	Number of Employees Directly in Contact with Clients	Number	(+) Outreach
			(-) Efficiency
**Total Assets**	Total Assets net of Allowances	Rupees	(+)
**Total Debt**	Both Subsidized & Commercial Debt Outstanding	Rupees	(-)
**Age**	Age since establishment	Years	(+)
**Operational Self-Sufficiency**	Operational Revenue/Financial & Operational Expenses	Ratio	(+)

### 3.2 Methodology

The efficiency of MFIs is usually measured via financial ratios, the most common ratios is non-interest expense to total revenue and staff expenditures to total revenue [13, 14, 48]. The smaller value of the ratios indicates the higher efficiency of the MFI. Serrano-Cinca et al. [47] argued that the use of any single ratio is problematic, especially in the context of MFIs. Efficiency is a multidimensional concept, and MFIs use more than a single input. Therefore, an MFI efficient in using one input may be inefficient in the use of others.

Besides the financial ratios, several other methods exist, such as Stochastic Frontier Analysis (SFA) and Distribution Free Approach (DFA), for measuring efficiency. However, DEA is one of the most used and reliable methods [49]. Traditionally, DEA is used to analyze the efficiency of nonprofit organizations (hospitals, for example). Still, it is equally valuable to measure efficiency in profit-oriented organizations [46]. The DEA is applicable over homogenous units, sharing the same inputs & outputs, and has the advantage to perform multiple comparisons simultaneously.

The DEA, a deterministic method, has a dominancy in microfinance research having several advantages. For instance, it is free from the frontier’s functional specification, suitable, and flexible to the industries with multiple products like the microfinance industry [50]. The DEA estimates the decision-making units' technical efficiency (DMUs) while ignoring the price efficiency, which requires pairwise price ratios of the input and output variables of all the DMUs. Moreover, the estimation of price efficiency requires information related to the input and output prices faced by the DMUs. However, Charnes et al. [51] argued that knowledge about input and output variables is usually not available, making DEA the most suitable efficiency measurement technique. Price efficiency is also suitable/relevant when the objective is to find out the least expensive production methods [52]. DEA's basic weaknesses are its sensitivity, to some extent, towards sample size, type, and size of data and error measurements. Such limitations sometimes lead to biased estimates derived through DEA [53]. However, the advantage of using the DEA method outweigh the disadvantage.

A fundamental disadvantage of using Stochastic Frontier Analysis (SFA) is its dependency on the specification of the production function of the MFIs. These functions are not only difficult but sometimes impossible to specify correctly. Further, unlike SFA, DEA's application does not require inputs & outputs to be measured in the same units and is free from distributional assumptions [54]. It is highly recommended in settings where cost and profit are inappropriate to gauge the entities' performance [55–57] and in small sample sizes [58]. Moreover, the method's comparability is well established in macro samples [59]. This study utilizes the DEA approach to measure the Overall Technical Efficiency (OTE) of the POs. The OTE is a firm's ability to maximize output with a given level of inputs [60]. The OTE is a suitable measure of efficiency provided that MFIs are operating at their optimum level [58]. The OTE can further be decomposed into Pure Technical Efficiency (PTE) and Scale Efficiency (ScE). The PTE reflects the potential of an MFI to utilize its resources in the best possible manner and avoid miss-utilization of inputs, while the optimal size of production can be measured with the help of ScE.

Further, the OTE compares the efficiency of an MFI with all other MFIs irrespective of their size., The PTE, on the other hand, compares efficiencies of the MFIs having an identical size [61]. In this paper, the financial and social efficiency of the POs has been investigated from three aspects. These are Overall Technical Efficiency (OTE); Constant Returns to Scale (CRS), Pure Technical Efficiency (PTE); Variable Returns to Scale (VRS) and Scale Efficiency (ScE); Operational Scale.

The super-efficiency (SpE) of the *i*^*th*^ MFI in *t*^*th*^ time obtained in the first step is regressed on the *j*^*th*^ institutional characteristic of the *i*^*th*^ MFI at time *t*. The following panel data specification would enable us to identify the relevant institutional characteristics of the POs/MFIs that influence their social and financial efficiency.

$${SpE}_{it}=\delta_{0}+\sum_{j=1}^{k}\sum_{i=1}^{n}\sum_{t=2005}^{2015}\beta_{jit}X_{jit}+\epsilon_{it}$$ (1)

In Eq (1), the β is a vector of the parameters of interest, and X is a vector that contains k-institutional characteristics of the POs. The selection of a panel data estimation technique depends on the specific distribution assumed for the error term (*ϵ_it_*), which is tested using the Hausman [62] specification test.

## 4 Results & discussion

### 4.1 Financial and social efficiency of the POs

The results show that during the period 2005–15, the sample POs were overall approximately 58% efficient. Similarly, based on Pure Technical Efficiency (PTE), the estimated efficiency is 75%. Further, based on Scale Efficiency (ScE), the combined efficiency is estimated equal to 55%. Note that we used the median as an average instead of mean to minimize the effect of potential outliers. For instance, if we use the mean, the average percentage of OTE based efficient POs will reduce to 51.26%, which may be underestimated due to the global market turbulence in the year 2011–12 of the microfinance industry [63]. Further, individually, the financial and social percentage of efficient POs was recorded as 27.3% and 29.6%, respectively, during 2005–15 based on OTE. Likewise, PTE based financially and socially efficient POs were approximately equal to 40% and 48%, respectively. The OTE, PTE, and ScE scores-based percentage of efficient POs for each year during 2005–15 are provided in Table 6. The percentage of socially efficient POs being greater than financially efficient POs has a positive and a negative aspect. On the positive side, this result implies that the POs are more concerned with their social mission rather than profitability. On the negative side, financial inefficiency may reduce POs' breadth and depth of outreach and their ultimate financial sustainability. Contrary to OTE and PTE based efficient POs, the percentage of ScE based financially and socially efficient POs are observed roughly similar (42% and 41%, respectively).

**Table 6:** The percentage of efficient POs based on OTE (CRS), PTE (VRS) and scale efficiency.

Years	No of POs	OTE (%)			PTE (%)			ScE (%)			RTS
		FE	SE	OE	FE	SE	OE	FE	SE	OE	
**2005**	14	35.71	57.14	64.28	50	71.42	78.57	64.29	64.29	78.57	CRS
**2006**	15	33.33	46.67	60.00	40	73.33	80.00	53.33	46.67	53.33	DRS
**2007**	14	21.42	50	64.28	57.14	64.28	78.57	35.71	50.00	64.28	DRS
**2008**	19	36.84	47.39	68.42	42.10	63.15	78.94	63.16	42.10	73.68	DRS
**2009**	19	21.05	26.32	52.63	31.57	36.84	63.15	36.84	31.59	42.10	DRS
**2010**	19	36.84	26.31	57.89	63.15	47.36	78.94	42.11	36.84	63.15	DRS
**2011**	20	30.00	50.00	65.00	35.00	60.00	75.00	60.00	75.00	55.00	DRS
**2012**	30	3.00	20.00	23.33	40.00	43.33	60.00	13.33	33.33	30.00	DRS
**2013**	33	27.27	18.18	33.33	42.42	42.42	54.54	60.61	24.24	57.58	DRS
**2014**	35	14.28	17.14	30.30	34.28	40.00	60.00	31.42	25.71	40.00	DRS
**2015**	27	22.22	29.62	44.44	33.33	48.15	66.67	33.33	40.74	48.14	DRS
**Median**		27.27	29.62	57.89	40.0	48.15	75.0	42.11	40.74	55.0	

**Note:** FE stands for financially efficient, SE for Socially Efficient, OE for Overall Efficient, NIRS for Non-Increasing Returns to Scale, and RTS for Returns to Scale.

**Fig 1:**
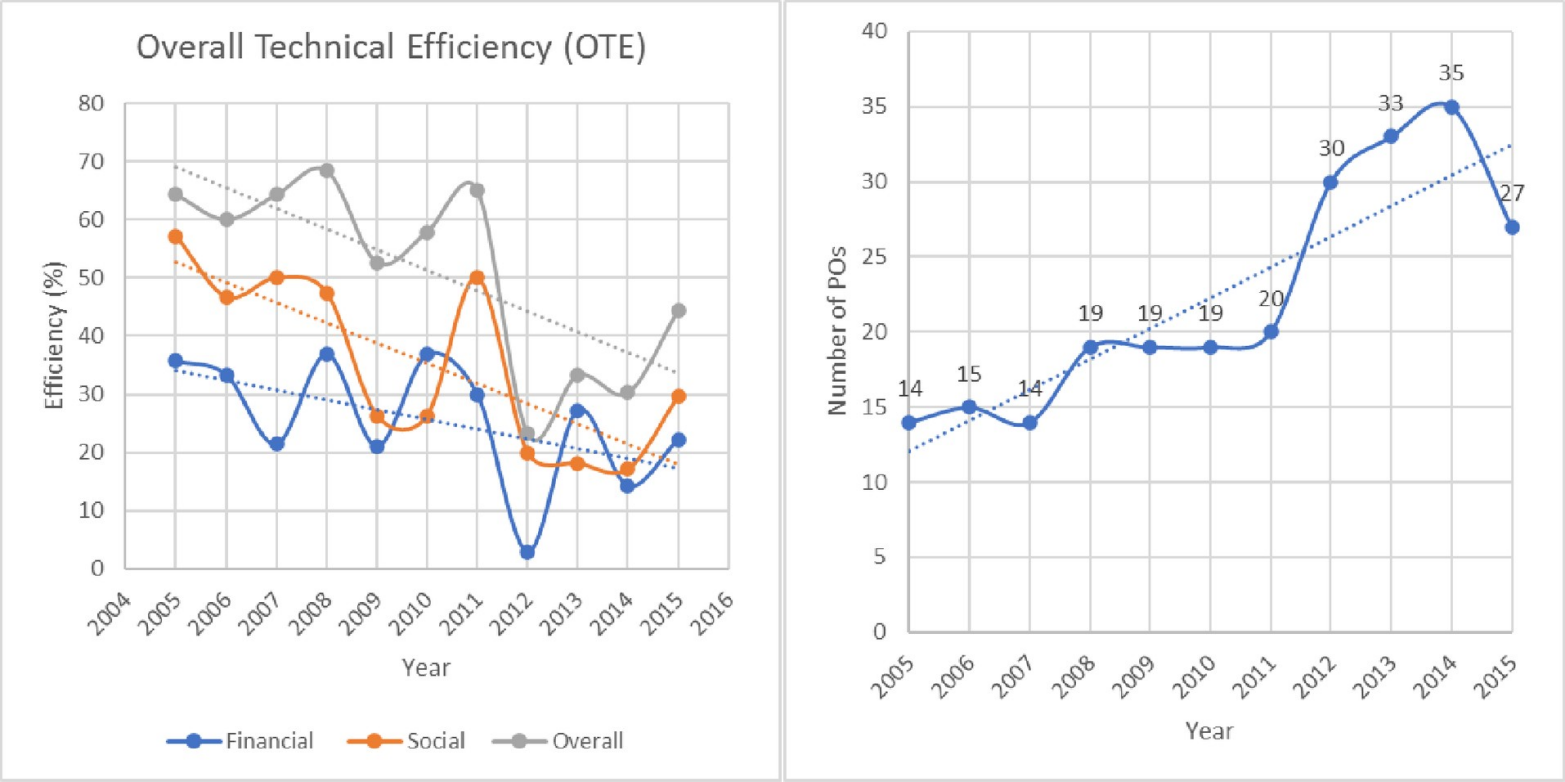
Time plots of (a) the percentage of efficient POs and (b) the number of Pos with the linear trend.

Further, to investigate how the proportion of efficient POs varies over time, the data were plotted against the years. Fig 1A is the time plot for financially, socially, and overall (combined financially and socially) percentage of efficient POs. A clear downward linear trend from graphical representation may be observed in the OTE based efficiencies (See Fig 1A). Various factors may be identified for the decrease in these efficiencies. The most common reason may be the period under investigation (2005–2015). It can be observed that the efficiency scores are at their highest at the beginning, which are years immediately before the financial crises of 2008–09 (See Littlefield and Kneiding [64] for evidence that MFIs are not shock-resistant to financial crises.). The POs/MFIs were recovering from those crises when the financial turbulence of 2011–12 again had its negative impact on the efficiency scores of the POs. Since recoveries of the MFIs occur with a time lag [35], the negative trend in the efficiency scores may be attributed to the impact of the two crisis periods. Another reason may be due to an increase in the number of new POs with respect to time. Fig 1B shows graphically how the number of POs increasing throughout the period 2005–15.

Further, from Fig 1A, the financial, social, and overall (combined) efficiency curves of the POs are at their minimum for the financial year 2011–12. The OTE based financial, social, and overall (combined) percentage of efficient POs for the financial year 2011–12 were only 3%, 20%, and 23.3%, respectively. The financial efficiency was recovered in the following years. However, the social efficiency was in further decrease for the next couple of years (until 2014) and then recovered to some extent in 2015. This finding is also standard as MFIs are expected to focus more on financial efficiency, as opposed to social efficiency during the after-crises recovery period [35]. This assertion is also supported because financial efficiency scores are more volatile than social efficiency scores in Fig 1A. Further, the OTE based financial efficiency was mostly lower than social efficiency throughout the period 2005–15. In contrast, the overall (combined) efficiency remained greater throughout 2005–15 (See Fig 1A).

**Fig 2:**
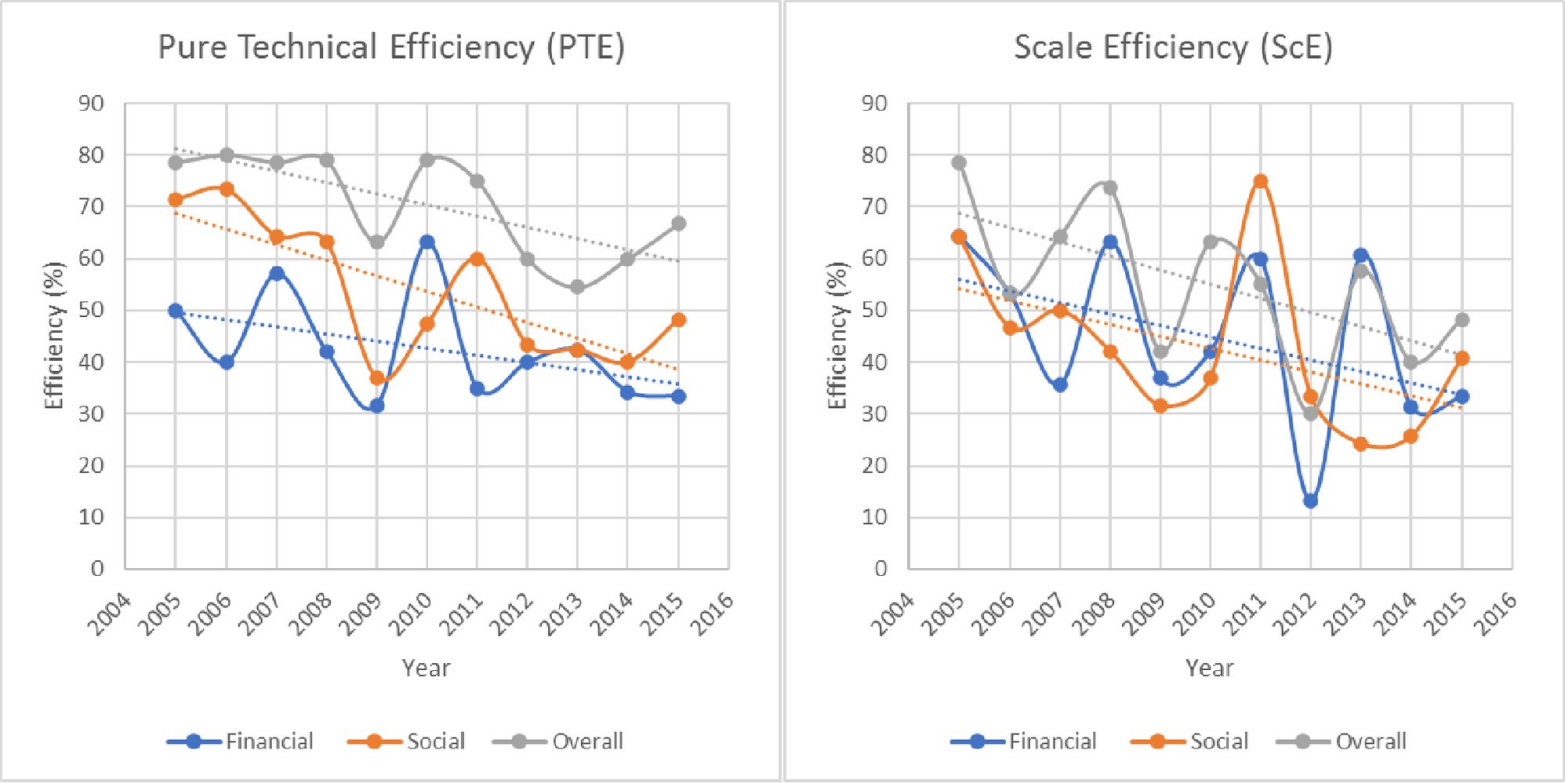
Time plot of the percentage of efficient POs during 2005–2015.

Similarly, Pure Technical Efficiency (PTE) and Scale Efficiency (ScE) based POs efficiencies were investigated for the period 2005–15. Fig 2 contained two plots representing data visualization for PTE and ScE based percentage of efficient POs. A linear decreasing trend may be observed in all three curves of financial, social, and overall (combined) PTE and ScE based efficient POs. The PTE based socially efficient POs are decreasing with a higher rate of change as compared to financially efficient POs (see Fig 2A). In contrast, the rate of decrease in ScE based financially and socially efficient POs are roughly identical since the trend lines are roughly parallel by visual inspection of Fig 2B. Further, from the curves' pattern, a negative serial correlation may be determined from the data. This shows that current-year financial and social efficiencies negatively affect the efficiencies of POs/MFIs during the next year. Time series modeling of the data will be interesting; however, that is beyond this paper's scope.

Further, from Fig 2A, based on the PTE scores, the POs were socially more efficient than financial efficiency from 2005 to 2015 except for the year 2010, in which the POs were financially more efficient. Further, from the graphs in Figs 1 and 2, it is observed that considering whatever efficiency score amongst OTE, PTE, or ScE, the POs are more overall efficient than social and financial. This, however, may be due to the sensitivity of the DEA method to the number and type of inputs selected in the three specifications [19]. Further, social efficiency is almost consistently greater than financial efficiency based on OTE and PTE. In contrast, based on the ScE scores, the social and financial efficiency of the POs reciprocated each other over time. For example, particularly in the years 2008 and 2013, the percentage of financially efficient POs was considerably higher than socially efficient POs. However, in the years 2007, 2011, 2012, and 2015, the socially efficient POs were greater than financially efficient POs.

Besides the above important results, and in line with previous research, for example [65, 66], we also found that relaxing the assumption of CRS causes average efficiency scores of the POs increase under the VRS assumption. The results further revealed that, except for the year 2005, the POs were operating at the stage of Decreasing Return to Scale (DRS) over the study period and hence are unable to reap the scale benefits/economies.

### 4.2 Institutional characteristics of the POs and efficiency

Descriptive statistics of the variables used for investigating the covariates of the financial and social efficiency of the POs are given in Table 7 below. Note that all variables are log-transformed and then mean-centered (except super efficiency) to reduce collinearity and the impact of outliers for use in the regression specifications.

**Table 7:** Summary statistics of the data.

Variable	Overall Mean	Panel Property	Std. Deviation	Minimum	Maximum
Super Efficiency	4.90	Overall	0.66	3.07	6.80
		Between	0.51	4.09	6.25
		Within	0.41	3.52	6.06
Age of the MFI	2.51	Overall	0.58	0.01	3.36
		Between	0.50	1.09	3.17
		Within	0.28	1.18	3.26
Operational Self-Sufficiency	4.72	Overall	0.54	2.86	8.93
		Between	0.30	3.33	5.15
		Within	0.49	3.24	8.81
No. of Branches	3.18	Overall	1.37	0.69	6.66
		Between	1.26	0.69	6.06
		Within	0.49	1.22	5.24
Personnel	5.32	Overall	1.45	1.61	8.22
		Between	1.35	2.68	7.89
		Within	0.49	3.45	7.55
Loan Officers	4.48	Overall	1.56	1.09	8.01
		Between	1.42	1.79	7.52
		Within	0.58	2.37	6.70
Total Assets	13.28	Overall	1.59	10.04	17.33
		Between	1.42	10.42	16.19
		Within	0.69	10.76	16.17
Total Debt	12.62	Overall	1.91	6.23	16.17
		Between	1.73	7.70	15.54
		Within	0.92	8.44	17.65

Table 8 below contains the model fit and diagnostic results. The results show cross-sectional heteroskedasticity, requiring estimation with robust standard errors in the model. The panel heterogeneity tests (both cross-section and period) imply cross-sectional and period effects. Hence, a fixed effect estimation relative to pooled regression would be more appropriate. Likewise, the cross-sectional dependence test results also imply that disturbances are cross-sectionally interdependent, and hence estimation based on random effect models may be problematic [67]. However, the results obtained from the Hausman [62] test imply that random effect modeling would be more appropriate than fixed effect modeling. Endogeneity of all the institutional characteristics is tested using Durbin-Wu-Hausman (DWH) test and by estimating covariance between residual term and individual, institutional characteristics. None of the institutional characteristics are, however, found to be endogenous. It is worth noticing that Eq (1) is estimated with both random and fixed effects approaches to account for the implications of all the diagnostic tests. The model fit results are given in the upper panel of Table 8 below.

**Table 8:** Intuitional characteristics of the POs and super efficiency (white cross-sectional robust standard errors).

Variables	Coefficients			Endogeneity Tests	
	CS-RE	CS-FE	TW-FE	Cov(eX)	DWH
Age of the POs	0.131 (0.014)	0.110 (0.000)	0.045 (0.551)	-0.000 (0.821)	0.021 (0.939)
Operational Self-Sufficiency	0.081 (0.058)	0.086 (0.009)	0.071 (0.038)	-0.008 (0.266)	0.989 (0.069)
Number of Branches	-0.054 (0.554)	-0.045 (0.598)	-0.042 (0.608)	-0.011 (0.160)	-0.591 (0.167)
Personnel	0.300 (0.023)	0.309 (0.031)	0.343 (0.018)	-0.009 (0.199)	-0.057 (0.902)
Loan Officers	-0.251 (0.000)	-0.258 (0.000)	-0.283 (0.000)	0.001 (0.843)	-0.057 (0.821)
Total Assets	-0.219 (0.000)	-0.203 (0.000)	-0.243 (0.000)	-0.011 (0.296)	0.202 (0.454)
Total Debt	0.076 (0.000)	0.063 (0.009)	0.063 (0.002)	0.010 (0.497)	0.064 (0.541)
Constant	4.508 (0.000)	4.508 (0.000)	4.506 (0.000)		
Adjusted R2	0.168	0.549	0.629		
F-Stats	10.842 (0.000)	12.303 (0.000)	10.876 (0.000)		
**Diagnostic Tests**					
*Panel Heteroskedasticity LR Test (Cross-Section)*				194.8013 (0.0000)	
*Panel Heteroskedasticity LR Test (Period)*				28.3306 (0.6989)	
*Panel Heterogeneity F Test (Cross-Section)*				11.6822 (0.0000)	
*Panel Heterogeneity F Test (Period)*				2.7358 (0.0031)	
*Cross-Section dependence Test (Breusch-Pagan LM)*				1070.243 (0.000)	
*Hausman Test*				9.6557 (0.2089)	

Results obtained in this study are generally in line with the previous research, for instance [10, 16, 25, 26, 28, 36, 68]. The age of the PO has a positive and statistically significant impact on their super-efficiency in two out of the three estimated specifications. Similarly, the efficiency of the POs is also positively and significantly affected by the personnel. Recent empirical research, such as Riaz and Gopal [36], has generally reported similar findings concerning the performance determinants of Pakistan based MFIs.

Total debt in this study reflects the importance of capital structure decisions on POs' performance. Starting with Miller and Modigliani's "irrelevance hypothesis" back in 1958, whether capital structure decisions influence a firms' performance is yet to be empirically solved. For instance, and in the context of the microfinance industry, Pandey and Sinha [31] reported debt to equity ratio being positively influencing MFI's performance while studies [25, 69–73] found the two variables to be negatively correlated. However, our study's findings clearly show that total debt has a positive and significant relationship with efficiency in all three estimated specifications.

A similar result also emerges concerning the impact of operational self-sufficiency on efficiency. Our results show that operational self-sufficiency contributes positively and significantly to the super efficiency of the sample POs/MFIs. Prior research on the association of operational self-sufficiency and efficiency has reported mixed results. For instance, studies [28, 74] reported a trade-off between operational self-sufficiency and social efficiency. In contrast, recent literature from Pakistan has reported it otherwise (for example [17, 39]). Further, most of the recent studies, for example [32, 35], have reported a positive correlation between financial considerations and efficiency.

This study's most pivotal result is the impact of POs size, measured by total assets, on super efficiency. Most recent indigenous studies [16, 37, 40] report a positive relationship between size and MFIs performance. Our results, however, show that total assets and super efficiency of POs are negatively and significantly related. However, this result is more in line with international evidence [25, 27, 29], except the results reported in study [21]. Similarly, the number of loan officers in POs also contributes negatively to their super efficiency. According to (Hina [73]: 255 & 274), the number of loan officers does not necessarily translate into more productivity and maybe negatively contributing to the efficiency of an MFI.

## 5 Conclusions and policy implications

Since its outset, microfinance has replaced many of the old poverty alleviation programs around the world. Besides its other contrasts with the old poverty alleviation programs, contemporary MFIs performs the dual objectives of poverty eradication in a self-sustainable way and hence relies very little on government support and subsidies. However, Pakistan's situation is different from the rest of the world, where most of the MFIs are still heavily dependent on government grants, subsidies, and soft loans provided via the Pakistan Poverty Alleviation Fund (PPAF). Since public funds are involved, and that the prevailing pandemic is surely pressing the meager availability of public funds, it is high time to review the performance of partner organizations of the PPAF. With this objective upfront, the present study reviewed the financial and social performance—and its determinants—of the POs of the PPAF. Penal data for the period of (2005–2015) has been collected from the database of the Mix-Market and Pakistan Microfinance Network. Using the DEA approach, the efficiency of the POs has been analyzed in the light of CRS, VRS, and with respect to the operational scale. The model fit results show that age, Operational Self-Sufficiency, personnel, loan officers, assets, and debt statistically affect the partner organizations' efficiencies. The diagnostic tests for endogeneity, heteroskedasticity, heterogeneity, and cross-sectional dependence were performed to make the results reliable.

The summary statistics and data visualization show that socially efficient POs were greater in percentage than the financially efficient POs based on the OTE and PTE scores. In contrast, based on the ScE scores, socially and financially efficient POs were found roughly identical in percentage except for a few years in 2005–15. Further, from the time plot of the percentage of efficient POs, a downward trend was observed. However, the decay rate observed (visually) in financially and socially efficient POs differed except for the score obtained via the ScE, where the rate of decrease in percentage observed in the two scores was roughly identical. Moreover, from visual inspections of the graphs, a negative serial correlation is also identified. As a result, it may be concluded that the current-year efficiency has a negative effect on the POs efficiency in the next year.

The second stage analysis implemented Panel Data Regression Technique to know the relative importance of the institutional characteristics of the POs in determining their social and financial efficiency. Concerning the institutional determinants of the POs performance, age of POs, Operational Self-sufficiency, Personnel, and the Total debt have a positive and statistically significant effect. In contrast, Loan Officers and total assets have a negative and significant effect on efficiency. Interestingly, the number of branches has an insignificant effect on POs performance.

This study's findings have important policy implications for the way MFIs operate and are regulated in Pakistan. At the operations level, the four statistically significant inputs (Personnel, loan officers, total assets, and total debt) are opposite in sign. Hence, the POs must reconsider the optimal input mix. More specifically, the efficiency of the POs of the PPAF requires POs to increase their number of staff other than loan officers and to finance their operations through debt rather than equity. The regulator's (PPAF) sole objective of eliminating poverty and empowering women can best be achieved by financing projects executed by more efficient POs. POs smaller in size (as proxied by total assets) and bigger in age contribute positively to efficiency. PPAF can more rigorously achieve its objectives by allocating more funds to smaller but older POs.

Operational self-sufficiency being positively influencing efficiency implies that the PPAF must reconsider how it regulates POs concerning the markup they charge on microcredit. Given the operational costs, operational revenues are directly proportional to markup rates. Therefore, any stringent rules requiring POs to follow markup ceilings necessarily compromise operational self-sufficiency and hence the efficiency of the POs. Hence to ensure greater efficiency, the PPAF must allow POs the flexibility to adjust their markup rates within certain limits. It can also encourage POs to acquire more debt by deducting interest on the debt from their corporate income tax liabilities.

## Supporting information

S1 Data (XLSX)
